# Supplementary material for: Multiscale relevance of natural images
Source: Sci Rep. 2023 Sep 9;13:14879. doi: 10.1038/s41598-023-41714-0 (PMC10492821; doi:10.1038/s41598-023-41714-0)
Supplement: Supplementary file 1 — Supplementary Information. [file 41598_2023_41714_MOESM1_ESM.pdf]

Supplementary Material

April 11, 2023

A Generation of $1/f^\alpha$ Gaussian fields

The $1/f^\alpha$ textures used in Sec. A are generated through a filtering procedure. Consider a field $\phi(\boldsymbol{r})$ of given autocorrelation function $C(\boldsymbol{r})$. Using the convolution theorem one has $\tilde{C}(\boldsymbol{q}) = |\tilde{\phi}(\boldsymbol{q})|^2$ in Fourier space with $|\boldsymbol{q}| = f$. A natural way to generate a random Gaussian field ϕ with prescribed correlations is:

$$\phi = \mathcal{F}^{-1}\left(\sqrt{|\tilde{C}(\boldsymbol{q})|}\tilde{\eta}(\boldsymbol{q})\right), \tag{1}$$

where $\eta(\boldsymbol{r})$ is a Gaussian white noise. Note that, as a result, the textures display periodic boundary conditions. Also note that the H = 0 (equivalently $\alpha = 1$) case corresponds to logarithmic spatial correlations of the form (see e.g. M. Peskin, An introduction to quantum field theory (CRC press, 2018)):

$$C(\boldsymbol{r}) = -\lambda\left(\log\frac{r}{\xi} + \log 2 - \gamma\right), \tag{2}$$

where $r = |\boldsymbol{r}|$, ξ^{-1} is a regularizing constant that can be interpreted as a low frequency cutoff, and γ is the Euler constant.
